# Hospital Rurality and Gene Expression Profiling for Early-Stage Breast Cancer among Iowa Residents (2010–2018)

**DOI:** 10.1155/2022/8582894

**Published:** 2022-08-30

**Authors:** Danielle Riley, Mary Charlton, Elizabeth A. Chrischilles, Ingrid M. Lizarraga, Sneha Phadke, Brian J. Smith, Adam Skibbe, Charles F. Lynch

**Affiliations:** ^1^Department of Epidemiology, College of Public Health, University of Iowa, Iowa City, Iowa, USA; ^2^Department of Surgery, University of Iowa Hospitals and Clinics, Iowa, Iowa City, USA; ^3^Department of Internal Medicine-Hematology, Oncology and Blood and Marrow Transplantation, University of Iowa Hospitals and Clinics, Iowa, Iowa City, USA; ^4^Holden Comprehensive Cancer Center, University of Iowa, Iowa, Iowa City, USA; ^5^Department of Geographical and Sustainability Sciences, College of Liberal Arts and Sciences, University of Iowa, Iowa, Iowa City, USA

## Abstract

**Objective:**

Given the challenges rural cancer patients face in accessing cancer care as well as the slower diffusion and adoption of new medical technologies among rural providers, the aim of our study was to examine trends in gene expression profiling (GEP) testing and evaluate the association between hospital rurality and receipt of GEP testing.

**Methods:**

Data from the Iowa Cancer Registry (ICR) were used to identify women with newly diagnosed, histologically confirmed breast cancer from 2010 through 2018 who met eligibility criteria for GEP testing. Patients were allocated to the hospitals where their most definitive surgical treatment was received, and Rural-Urban Commuting Area codes were used to categorize hospitals into urban (*N* = 43), large rural (*N* = 16), and small rural (*N* = 48). Adjusted odds ratios (aORs) and 95% confidence intervals (CIs) were estimated using multivariable logistic regression to evaluate the association between hospital rurality and GEP test use, adjusting for demographic and clinical characteristics. The association between test result and treatment received was assessed among patients who received Oncotype DX (ODX) testing.

**Results:**

Of 6,726 patients eligible for GEP test use, 46% (*N* = 3,069) underwent testing with 95% receiving ODX. While overall GEP testing rates increased over time from 42% between 2010 and 2012 to 51% between 2016 and 2018 (*P*_trend_ < 0.0001), use continued to be the lowest among patients treated at hospitals in small rural areas. The odds of GEP testing remained significantly lower among patients treated at hospitals located in small rural areas (aOR 0.55; 95% CI 0.43–0.71), after adjusting for demographic and clinical characteristics. ODX recurrence scores were highly correlated with chemotherapy use across all strata of hospital rurality.

**Conclusions:**

GEP testing continues to be underutilized, especially among those treated at small rural hospitals. Targeted interventions aimed at increasing rates of GEP testing to ensure the appropriate use of adjuvant chemotherapy may improve health outcomes and lower treatment-related costs.

## 1. Introduction

In the United States, breast cancer accounts for 31% of all cancers diagnosed among females (excluding nonmelanotic skin cancers), with an estimated 287,850 new cases expected in 2022 [1]. Hormone receptor-positive (HR+) and human epidermal growth factor receptor 2 negative (HER2−) breast cancer are the most common subtypes, accounting for approximately 70% of all breast cancer cases in the United States. Of those, approximately 75% are axillary lymph node negative (LN−) [2]. For patients diagnosed with HR + tumors, the use of endocrine therapy following surgery (mastectomy or breast-conserving surgery (BCS)) with or without radiation therapy is considered the standard of care. To further reduce the overall risk of recurrence, adjuvant chemotherapy may also be recommended [3].

Historically, treatment decisions related to the use of adjuvant chemotherapy have been made using clinicopathologic characteristics (i.e., age at diagnosis, tumor size, and tumor grade) in combination with the treatment preferences of the patient and clinician(s). However, studies suggest approximately 85% of patients diagnosed with HR+, LN− breast cancer are unlikely to derive significant benefit from the addition of chemotherapy given the low 5-year rate of recurrence associated with endocrine therapy alone along with pathologic features that can make these tumors less responsive to traditional cytotoxic chemotherapy [4, 5]. Furthermore, chemotherapy is usually accompanied by significant adverse side effects including nausea, fatigue, hair loss, neuropathy, and cognitive dysfunction. Approximately 60% of early-stage breast cancer patients treated with adjuvant chemotherapy report experiencing at least one severe side effect of treatment, and 19% of those require unscheduled care through emergency room visits and/or inpatient admissions [6].

To better identify those at high risk for recurrence who would benefit from the addition of adjuvant chemotherapy and minimize avoidable side effects associated with overtreatment among patients with a low risk of recurrence, several GEP assays have been developed including Oncotype DX (ODX; Genomic Health, Inc., Redwood, CA), MammaPrint (Agendia, Inc., Amsterdam, The Netherlands), Predictor Analysis of Microarray 50 (Prosigna (PAM50); NanoString Technologies, Seattle, WA), EndoPredict (Myriad Genetics, Salt Lake City, UT), and the Breast Cancer Index (BCI; Biotheranostics, Inc., San Diego, CA) [7]. Studies suggest breast cancer treatment decisions guided by GEP test results reduce chemotherapy recommendations by approximately one-third, highlighting the clinical utility of such tests [8–10].

The use of GEP tests for select patients with early-stage breast cancers is included in clinical practice guidelines published by the National Comprehensive Cancer Network (NCCN) [3] and the American Society for Clinical Oncology (ASCO) [11]. Though research demonstrates the overall cost-effectiveness of GEP tests [12, 13], recent studies suggest fewer than 50% of eligible patients undergo testing [14–18], with disparities in testing observed among racial minorities and those of low socioeconomic status [19–22].

Patients residing in rural areas commonly face challenges in accessing cancer care due in part to increased travel burden, limited availability of oncology specialists, and reduced availability of targeted treatments that may be informed by GEP testing [23–26], which can result in poorer quality of care and health outcomes [27, 28]. Rural providers may also face barriers in the delivery of evidence-based care driven by the slower adoption of new medical technologies and nonadherence to the latest clinical practice guidelines potentially resulting in the use of suboptimal or inadequate treatment regimens [29–33]. Increasing the proportion of eligible breast cancer patients, including those treated in rural areas, who undergo GEP testing and receive appropriate adjuvant chemotherapy could result in better health outcomes and lower treatment costs [34]. The role of rurality has rarely been evaluated in studies examining determinates of GEP test use [20, 21, 35–37]. Therefore, the aim of this study was to describe temporal trends and examine rural-urban differences in GEP testing among women diagnosed with early-stage, HR+, HER2−, and LN− breast cancer in Iowa and among women who received ODX, determining how the results are associated with chemotherapy use.

## 2. Materials and Methods

A secondary data analysis of female breast cancer patients was performed using data extracted from the ICR. The ICR is a population-based registry that routinely collects information on all newly diagnosed cancers in Iowa and includes patient demographics, tumor characteristics, first course of therapies, and survival data. The ICR has been a member of the National Cancer Institute's Surveillance, Epidemiology, and End Results (SEER) program since 1973 [38].

Given the deidentified nature of the data, the Institutional Review Board at the University of Iowa determined that this study did not meet the criteria for human subjects' research and was exempted from further review.

### 2.1. Study Population

A total of 6,821 female Iowa residents, aged 20 years and older with histologically confirmed, unilateral, first primary, breast cancer diagnosed between January 1, 2010 and December 31, 2018, who met eligibility criteria for GEP testing defined as stage I/II (AJCC 6^th^ or 7^th^ edition), HR+ (defined as estrogen receptor-positive and/or progesterone receptor-positive), HER2−, LN− breast cancer measuring >0.5 cm were identified by the ICR. Patients were excluded if they had not undergone surgical resection of their tumor via mastectomy or lumpectomy (*N* = 7). Patients with missing surgical facility were also excluded (*N* = 88).

### 2.2. Study Measures

#### 2.2.1. Defining GEP Testing

Receipt of GEP testing, including ODX, MammaPrint, EndoPredict, BCI, and Prosigna, was obtained from the ICR. Given the differences in scoring methods as well as the incomplete ascertainment of all GEP test results prior to 2018, ODX recurrence scores (RS), which accounted for 95% of our GEP-tested population, were the focus of our subsequent analyses. For patients with ODX testing, RS was categorized as low (RS < 18), intermediate (RS 18–30), or high (RS ≥ 31) risk. Considering the study time frame, the RS categories were chosen based on the validation study conducted by Paik et al. [39]. Patients with no GEP testing information were considered as not receiving ODX.

#### 2.2.2. Defining Adjuvant Chemotherapy

Adjuvant chemotherapy was defined as the first course of treatment initiated at any facility following surgical resection of the tumor. Patients were considered to have received adjuvant chemotherapy regardless of the type of agent, number of agents, or duration of therapy.

#### 2.2.3. Defining Rurality

**Figure 1:**
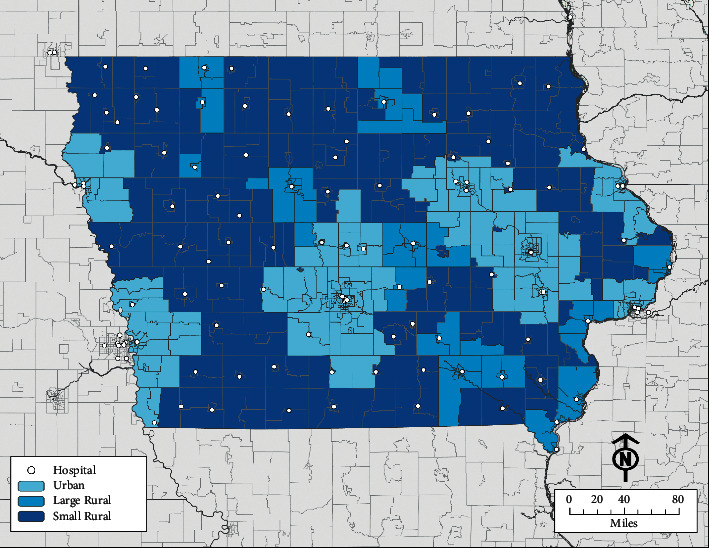
Iowa Rural-Urban Commuting Area (RUCA) designations.

Rural-Urban Commuting Area (RUCA) codes [40], based on data from the 2010 decennial census, were used to categorize the level of rurality for each patient as well as the hospital that administered the most definitive surgical treatment for each patient [41]. Urban areas included RUCA codes 1.0, 1.1, 2.0, 2.1, 3.0, 4.1, 5.1, 7.1, 8.1, and 10.1; large rural areas (i.e., towns with populations between 10,000 and 49,999 with daily commuting flows of 10% or more to urban areas with populations of 50,000 or more) included codes 4.0, 4.2, 5.0, 5.2, 6.0, and 6.1; and small/isolated rural areas (i.e., towns with populations fewer than 10,000 with less commuting connectivity to urban areas) included codes 7.0, 7.2, 7.3, 7.4, 8.0, 8.2, 8.3, 8.4, 9.0, 9.1, 9.2, 10.0, 10.2, 10.3, 10.4, 10.5, and 10.6. Among hospitals serving Iowans, 43 were categorized as urban, 16 as large rural, and 48 as small rural. Census-tract RUCA designations within Iowa are presented in Figure 1.

#### 2.2.4. Demographic and Clinical Characteristics

Patient-level demographic and clinical characteristics obtained from the ICR included age at diagnosis, race/ethnicity, marital status, insurance status, year of diagnosis, cancer stage (based on the derived AJCC stage group; 6^th^ or 7^th^ edition), histology, tumor size, and tumor grade. Insurance status was defined as insured (private or Medicare), Medicaid coverage (any Medicaid, including Indian Health Service), and uninsured. Patients with no known insurance coverage at the time of diagnosis (*N* = 145) were assigned to the uninsured category. Census-tract-level median household income derived from the 2017 American Community Survey was included [42].

### 2.3. Statistical Analysis

Pearson chi-square tests were used to examine the unadjusted associations between categorical variables and hospital rurality as well as GEP testing. Cochran-Mantel-Haenszel tests were used to examine trends among ordinal variables. Variable selection was performed *a priori* based on the Andersen-Newman framework [43] as well as existing literature. A multivariable logistic regression model was used to evaluate the association between hospital rurality and GEP testing, adjusting for demographic and clinical characteristics. As a result of high multicollinearity with tumor size, cancer stage was removed from the final model. Results are presented as adjusted odds ratios (aORs) using a 95% CI. Categorical variables are reported as frequencies and percentages. We used SAS software, version 9.4 (SAS Institute Inc., Cary, North Carolina) and considered two-sided *P*-values <0.05 to be statistically significant.

## 3. Results

Characteristics of the study population by hospital location are presented in Table 1. Patients treated at hospitals located in small rural areas were more often older at the time of diagnosis, unmarried, uninsured, and residing in small rural areas as well as areas with lower median household income. The proportion of patients treated at hospitals in small rural areas decreased between 2010 and 2012 and 2016 and 2018. Compared to patients treated at hospitals located in urban or large rural areas, patients treated at hospitals in small rural areas were more frequently diagnosed with stage II tumors, measuring 2.0–4.0 cm. There were no clinically meaningful differences in hospital location by tumor histology or grade.

Patient demographic and clinical characteristics by residential location are presented in Table 2. Patients residing in census-tracts designated as “small rural” were more often older at the time of diagnosis and have lower median household income. Additionally, patients residing in small rural areas were more frequently diagnosed with stage II tumors, measuring 2.0–4.0 cm. Finally, compared to urban patients, patients residing in small rural areas less often received GEP testing despite eligibility. There were no clinically meaningful differences between residential locations by tumor histology or grade.

Of 6,726 patients eligible for GEP test use based on national clinical practice guidelines, 46% (*N* = 3,069) underwent testing. Patients treated at hospitals in small rural areas received testing significantly less often compared to those who underwent treatment at hospitals located in urban or large rural areas (small rural: 28.6% vs. large rural: 43.4% vs. urban: 47.2%; *P* < 0.0001). In addition, patients who were older at the time of diagnosis, unmarried, and residing in areas with lower median household income received testing less frequently. There were clinically meaningful differences in GEP test use by tumor histology, size, and grade (Table 3).

**Figure 2:**
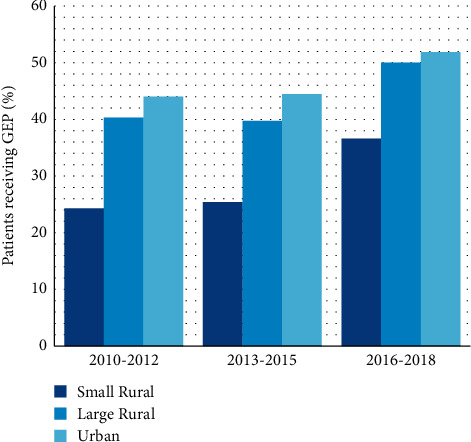
GEP testing trends among women with early-stage, HR+, HER2−, LN− breast cancer by hospital location. GEP, gene expression profiling; HER2−, human epidermal growth factor receptor 2 negative; HR+, hormone receptor-positive; LN−, lymph node negative.

While overall GEP testing rates increased over time from 42% between 2010 and 2012 to 51% between 2016 and 2018 (*P*_trend_ < 0.0001) (Table 3), use continued to be the lowest among patients treated at hospitals in small rural areas across all three study periods (Figure 2).

### 3.1. Use of GEP Tests

In univariable analysis, the odds of GEP testing were significantly lower among patients treated at hospitals located in large rural and small rural areas. In addition, the odds of GEP testing were lower among older patients and those who were unmarried at the time of diagnosis. The odds of GEP testing increased with increasing median household income, increased over time, and increased with increasing tumor grade. The odds of GEP testing varied by tumor histology and tumor size (Table 4). In multivariable analysis, the odds of GEP testing remained significantly lower among patients treated at hospitals located in small rural areas (aOR 0.55; 95% CI 0.43–0.71) after adjusting for age, marital status, insurance status, median household income, year of diagnosis, tumor histology, tumor size, and tumor grade.

### 3.2. Receipt of Chemotherapy by ODX RS

ODX testing accounted for 95% of all GEP testing in our study population (Table 1). Of 2,914 patients who received ODX testing, 60% had a RS less than 18 (low risk), 32% had a RS between 18 and 30 (intermediate risk), and 8% had a RS of 31 or greater (high risk). Recurrence scores were highly correlated with receipt of adjuvant chemotherapy, regardless of hospital rurality (Table 5). Overall, adjuvant chemotherapy was administered to 1.7% of patients with a low RS compared to 83% of those with a high RS. Among patients with an intermediate RS, 29% received chemotherapy with this stratum showing the largest amount of variability between hospital locations.

## 4. Discussion

In this population-based study, fewer than half of all breast cancer cases who were eligible for GEP testing, based on national clinical practice guidelines, received it. Women eligible for GEP testing were much less likely to receive testing if they sought care at a hospital in a small rural area. These hospitals accounted for 45% of hospitals in the study area and treated 6% of the study population. Patients who received care at hospitals in larger rural areas, accounting for 15% of hospitals and 13% of the study population, were also less likely to receive testing although not after adjustment for demographic and clinical characteristics. Once tested, women were equally likely to receive adjuvant chemotherapy irrespective of hospital rurality.

Our results are consistent with other studies that report testing rates between 25% and 54% among eligible breast cancer patients [14–22, 35–37]. While overall rates of GEP test use increased over time, approximately 63% of eligible patients treated at small rural hospitals did not receive GEP testing in the most recent study period (2016–2018) compared to 48% of patients treated at urban hospitals, providing further evidence that GEP assays continue to be underused.

In multivariable analysis, results demonstrate that patients who undergo treatment at hospitals located in small rural areas are significantly less likely to receive GEP testing compared to those treated at urban hospitals, after adjusting for demographic and clinical characteristics. Our finding of low testing rates among patients treated at rural hospitals helps to explain previously reported associations between GEP testing and rural-urban residence [20, 21, 35–37] by delving further into where breast cancer patients receive care [20, 21, 44]. A cross-sectional study by Lynch et al. [44] found that women who reside in rural counties or receive care at critical-access hospitals are less likely to receive ODX testing. In addition, women in Iowa had the fourth lowest testing rate of all states. According to ASCO's most recent “2020 Snapshot: State of the Oncology Workforce in America”, only 11.6% of oncologists practice in rural areas, with 66% of rural counties having no practicing oncologist [45]. Such shortages of cancer specialists in rural communities may negatively affect GEP testing rates as most tests are ordered by medical oncologists [46]. Additionally, given the reduced availability of resources, rural hospitals are unable to offer multidisciplinary services including molecular tumor boards, which have been found to increase the likelihood of GEP testing [30, 47, 48].

Demographic and clinical characteristics are known to influence the use of GEP tests for women diagnosed with early-stage breast cancer. Consistent with prior studies [14, 15, 17, 20, 21], the odds of GEP test use were greater among patients under the age of 60 at the time of diagnosis, among those who were married or living with a partner, among those residing in areas with higher median household income, and among those diagnosed with tumors measuring 2.0–4.0 cm as well as those with higher grade tumors. These patient characteristics were associated with location of the treating hospital.

Similar to previous studies [14, 15], there was a high correlation between ODX recurrence score and receipt of adjuvant chemotherapy as recommended based on prespecified risk categories. The largest variation in adjuvant chemotherapy use between hospitals was observed among the intermediate risk group, with the lowest rate of chemotherapy use among patients treated at small rural hospitals [49]. This finding highlights the uncertainty regarding whether chemotherapy is beneficial for those with a mid-range recurrence score prior to the release of results from the Trial Assessing Individualized Options for Treatment (TAILORx) trial in 2018, which was designed to address these knowledge gaps [50]. Of those who did not receive GEP testing, chemotherapy rates were the lowest among patients treated at large rural and small rural hospitals. Consistent with our findings, there is evidence that rural hospitals, including those that are Prospective Payment System hospitals as well as those that serve as critical-access hospitals, provide less chemotherapy compared to urban hospitals [51]. The low ODX testing rates along with the low rates of chemotherapy administered to patients who did not receive ODX testing at hospitals located in small rural areas are likely to have resulted in higher rates of undertreatment among our study population.

This is the first population-based study to evaluate differences in GEP test use by hospital location among guideline-eligible breast cancer patients and, therefore, provides new evidence that patients treated at rural hospitals receive less GEP testing compared to those treated at urban hospitals. While previous studies have adjusted for rurality, they were not designed to examine if hospital rurality influenced the odds of GEP testing in women diagnosed with early-stage breast cancer. Additionally, this study was conducted using data from the ICR, which has been a member of the SEER Program since its inception in 1973. Since 1998, this registry has received and maintained annual Gold Standard certification by the North American Association of Central Cancer Registries (NAACCR) [52].

However, several limitations of this study warrant consideration. Our population included predominantly non-Hispanic white patients residing in Iowa at the time of diagnosis, so results may not be generalizable to those of other racial/ethnic groups or to those living in other geographic regions. In addition, patient-provider decision-making processes that may impact the use of GEP tests were not assessable. Furthermore, our study lacked comorbidity information as well as individual data on socioeconomic factors, including income that have been shown to be associated with GEP testing in prior studies. As such, caution should be taken when interpreting the observed association between GEP test use and census-tract data.

## 5. Conclusions

In summary, the use of GEP testing has increased significantly over time among Iowa's guideline-eligible breast cancer patients but is still low. Despite the clinical utility and cost-effectiveness of such tests, GEP tests continue to be used in less than half of eligible patients, especially among those treated at hospitals located in small rural areas. Further research is needed to examine patient, provider, and hospital characteristics associated with GEP test use, including patient and provider treatment preferences as well as the ability of the patient to adhere safely to a full chemotherapy regimen. Such research may inform the development of targeted interventions aimed at increasing rates of GEP testing in rural areas to ensure the appropriate use of adjuvant chemotherapy and improve health outcomes.

## Figures and Tables

**Table 1:** Patient characteristics by hospital location among women with early-stage, HR+, HER2−, LN− breast cancer.

	Hospital Location			*P* value
	Urban area	Large rural area	Small rural area	
	(*N* = 5488)	(*N* = 853)	(*N* = 385)	
	N (Column %)	N (Column %)	N (Column %)	
Rural-urban residence				
Urban	3839 (70.0)	6 (0.7)	14 (3.6)	**<0.0001** ^**a**^
Large rural	422 (7.7)	522 (61.2)	14 (3.6)	
Small rural	1227 (22.4)	325 (38.1)	357 (92.7)	
Age at diagnosis, years				
<50	832 (15.2)	92 (10.8)	20 (5.2)	**<0.0001** ^**b**^
50–59	1308 (23.8)	156 (18.3)	62 (16.1)	
60–69	1728 (31.5)	257 (30.1)	112 (29.1)	
≥70	1620 (29.5)	348 (40.8)	191 (49.6)	
Marital status at diagnosis				
Married/Living with partner	3664 (66.8)	522 (61.2)	230 (59.7)	**0.0003** ^**a**^
Other^c^/Unknown	1824 (33.2)	331 (38.8)	155 (40.3)	
Insurance status				
Insured^d^	4981 (90.8)	777 (91.1)	344 (89.3)	**0.029** ^**a**^
Any Medicaid^e^	309 (5.6)	55 (6.5)	18 (4.7)	
Uninsured/Unknown	198 (3.6)	21 (2.4)	23 (6.0)	
Median household income				
<$50,000	1235 (22.5)	348 (40.8)	114 (29.6)	**<0.0001** ^**b**^
$50,000-$59,999	1409 (25.7)	354 (41.5)	164 (42.6)	
$60,000-$69,999	1302 (23.7)	126 (14.8)	78 (20.3)	
≥$70,000	1542 (28.1)	25 (2.9)	29 (7.5)	
Year of diagnosis				
2010–2012	1524 (27.8)	263 (30.8)	140 (36.4)	**0.001** ^**b**^
2013–2015	1859 (33.9)	302 (35.4)	122 (31.7)	
2016–2018	2105 (38.4)	288 (33.8)	123 (31.9)	
Cancer stage				
I	4464 (81.3)	694 (81.4)	284 (73.8)	**0.001** ^**a**^
II	1024 (18.7)	159 (18.6)	101 (26.2)	
Histology				
Ductal	4142 (75.5)	626 (73.4)	290 (75.3)	**0.019** ^**a**^
Lobular	659 (12.0)	87 (10.2)	44 (11.4)	
Ductal + Lobular	219 (4.0)	51 (6.0)	9 (2.3)	
Mucinous	169 (3.1)	30 (3.5)	17 (4.5)	
NOS/Other	299 (5.4)	59 (6.9)	25 (6.5)	
Tumor size, cm				
0.6–1.9	4205 (76.6)	653 (76.6)	266 (69.0)	**0.013** ^**b**^
2.0–4.0	1123 (20.5)	177 (20.7)	108 (28.1)	
>4.0	160 (2.9)	23 (2.7)	11 (2.9)	
Tumor grade				
Well differentiated	2175 (39.6)	322 (37.8)	135 (35.1)	0.413^a^
Moderately differentiated	2394 (43.7)	378 (44.3)	187 (48.6)	
Poorly differentiated/Undifferentiated	814 (14.8)	138 (16.2)	58 (15.1)	
Unknown	105 (1.9)	15 (1.7)	5 (1.2)	
GEP method				
Oncotype DX	2445 (44.6)	364 (42.7)	105 (27.3)	**<0.0001** ^**a**^
Other^f^	144 (2.6)	6 (0.7)	5 (1.3)	
Test not done	2899 (52.8)	483 (56.6)	275 (71.4)	

Abbreviations: CM, centimeter; GEP, gene expression profiling; HER2−, human epidermal growth factor receptor 2 negative; HR+, hormone receptor-positive; LN−, lymph node negative; NOS, not otherwise specified. Bold values indicate significance at *α* = 0.05. ^*a*^*P*-values are based on Pearson chi-square test for categorical variables. ^*b*^*P*-values are based on Cochran-Mantel-Haenszel test for trend for ordered variables. ^c^ Divorced, separated, single, and widowed. ^d^ Private insurance and Medicare. ^e^ Indian Health Service. ^f^ MammaPrint, EndoPredict, BCI, and Prosigna. Bold values indicate significance at *α* = 0.05.

**Table 2:** Patient characteristics by residential location among women with early-stage, HR+, HER2−, LN− breast cancer.

	Residential location			*P* value
	Urban area	Large rural area	Small rural area	
	(*N* = 3859)	(*N* = 958)	(*N* = 1909)	
	N (Column %)	N (Column %)	N (Column%)	
Age at diagnosis, years				
<50	612 (15.9)	111 (11.6)	221 (11.6)	**<0.0001** ^**a**^
50–59	918 (23.8)	212 (22.1)	396 (20.7)	
60–69	1218 (31.6)	296 (30.9)	583 (30.5)	
≥70	1111 (28.7)	339 (35.4)	709 (37.2)	
Marital status at diagnosis				
Married/Living with partner	2527 (65.5)	612 (63.9)	1277 (66.9)	0.261^b^
Other^c^/Unknown	1332 (34.5)	346 (36.1)	632 (33.1)	
Insurance status				
Insured^d^	3503 (90.8)	863 (90.1)	1736 (90.9)	0.089^b^
Any Medicaid^e^	216 (5.6)	69 (7.2)	97 (5.1)	
Uninsured/Unknown	140 (3.6)	26 (2.7)	76 (4.0)	
Median household income				
<$50,000	688 (17.8)	384 (40.1)	625 (32.7)	**<0.0001** ^**a**^
$50,000-$59,999	726 (18.8)	376 (39.2)	825 (43.2)	
$60,000-$69,999	971 (25.2)	150 (15.7)	385 (20.2)	
≥$70,000	1474 (38.2)	48 (5.0)	74 (3.9)	
Year of diagnosis				
2010–2012	1071 (27.8)	291 (30.4)	565 (29.6)	0.065^a^
2013–2015	1301 (33.7)	344 (35.9)	638 (33.4)	
2016–2018	1487 (38.5)	323 (33.7)	706 (37.0)	
Cancer stage				
I	3172 (82.2)	773 (80.7)	1497 (78.4)	**0.003** ^**a**^
II	687 (17.8)	185 (19.3)	412 (21.6)	
Histology				
Ductal	2929 (75.9)	679 (70.9)	1450 (76.0)	**0.033** ^**b**^
Lobular	459 (11.9)	123 (12.8)	208 (10.9)	
Ductal + Lobular	147 (3.8)	49 (5.1)	83 (4.4)	
Mucinous	110 (2.9)	39 (4.1)	67 (3.5)	
NOS/Other	214 (5.5)	68 (7.1)	101 (5.2)	
Tumor size, cm				
0.6–1.9	3007 (77.9)	718 (75.0)	1399 (73.3)	**0.001** ^**a**^
2.0–4.0	744 (19.3)	207 (21.6)	457 (23.9)	
>4.0	108 (2.8)	33 (3.4)	53 (2.8)	
Tumor grade				
Well differentiated	1577 (40.9)	355 (37.1)	700 (36.7)	**0.049** ^**b**^
Moderately differentiated	1648 (42.7)	437 (45.6)	874 (45.8)	
Poorly differentiated/Undifferentiated	559 (14.5)	148 (15.4)	303 (15.9)	
Unknown	75 (1.9)	18 (1.9)	32 (1.6)	
GEP method				
Oncotype DX	1721 (44.6)	409 (42.7)	784 (41.1)	**0.009** ^**b**^
Other^f^	99 (2.6)	12 (1.2)	44 (2.3)	
Test not done	2039 (52.8)	537 (56.1)	1081 (56.6)	

Abbreviations: CM, centimeter; GEP, gene expression profiling; HER2−, human epidermal growth factor receptor 2 negative; HR+, hormone receptor-positive; LN−, lymph node negative; NOS, not otherwise specified. Bold values indicate significance at *α* = 0.05. ^*a*^*P*-values are based on Cochran-Mantel-Haenszel test for trend for ordered variables. ^*b*^*P*-values are based on Pearson chi-square test for categorical variables. ^c^ Divorced, separated, single, and widowed. ^d^ Private insurance and Medicare. ^e^ Indian Health Service. ^f^ MammaPrint, EndoPredict, BCI, and Prosigna. Bold values indicate significance at *α* = 0.05.

**Table 3:** Patient and hospital characteristics by GEP testing for early-stage, HR+, HER2−, and LN− female breast cancer.

	GEP Testing		*P* value
	Yes	No	
	(*N* = 3069)	(*N* = 3657)	
	*N* (Row %)	*N* (Row %)	
Rural-urban residence			
Urban	1820 (47.2)	2039 (52.8)	**0.013** ^**a**^
Large rural	421 (44.0)	537 (56.0)	
Small rural	828 (43.4)	1081 (56.6)	
Hospital location			
Urban	2589 (47.2)	2899 (52.8)	**<0.0001** ^**a**^
Large rural	370 (43.4)	483 (56.6)	
Small rural	110 (28.6)	275 (71.4)	
Age at diagnosis, years			
<50	582 (61.6)	362 (38.4)	**<0.0001** ^**b**^
50–59	933 (61.1)	593 (38.9)	
60–69	1052 (50.2)	1045 (49.8)	
≥70	502 (23.2)	1657 (76.8)	
Marital status at diagnosis			
Married/Living with partner	2156 (48.8)	2260 (51.2)	**<0.0001** ^**a**^
Other^c^/Unknown	913 (39.5)	1397 (60.5)	
Insurance status			
Insured^d^	2769 (45.4)	3333 (54.6)	0.055^a^
Any Medicaid^e^	196 (51.3)	186 (48.7)	
Uninsured/Unknown	104 (43.0)	138 (57.0)	
Median household income			
<$50,000	708 (41.7)	989 (58.3)	**0.001** ^**b**^
$50,000-$59,999	879 (45.6)	1048 (54.4)	
$60,000-$69,999	709 (47.1)	797 (52.9)	
≥$70,000	773 (48.4)	823 (51.6)	
Year of diagnosis			
2010–2012	811 (42.1)	1116 (57.9)	**<0.0001** ^**b**^
2013–2015	977 (42.8)	1306 (57.2)	
2016–2018	1281 (50.9)	1235 (49.1)	
Cancer stage			
I	2462 (45.2)	2980 (54.8)	0.188^a^
II	607 (47.3)	677 (52.7)	
Histology			
Ductal	2364 (46.7)	2694 (53.3)	**<0.0001** ^**a**^
Lobular	379 (48.0)	411 (52.0)	
Ductal + Lobular	148 (53.0)	131 (47.0)	
Mucinous	56 (25.9)	160 (74.1)	
NOS/Other	122 (31.9)	261 (68.1)	
Tumor size, cm			
0.6–1.9	2273 (44.4)	2851 (55.6)	**<0.0001** ^**b**^
2.0–4.0	725 (51.5)	683 (48.5)	
>4.0	71 (36.6)	123 (63.4)	
Tumor grade			
Well differentiated	1032 (39.2)	1600 (60.8)	**<0.0001** ^**a**^
Moderately differentiated	1498 (50.6)	1461 (49.4)	
Poorly differentiated/Undifferentiated	495 (49.0)	515 (51.0)	
Unknown	44 (35.2)	81 (64.8)	

Abbreviations: CM, centimeter; GEP, gene expression profiling; HER2−, human epidermal growth factor receptor 2 negative; HR+, hormone receptor-positive; LN−, lymph node negative; NOS, not otherwise specified. Bold values indicate significance at *α* = 0.05. ^*a*^*P*-values are based on Pearson chi-square test for categorical variables. ^*b*^*P*-values are based on Cochran-Mantel-Haenszel test for trend for ordered variables. ^c^ Divorced, separated, single, and widowed. ^d^ Private insurance and Medicare. ^e^ Indian Health Service.

**Table 4:** Odds of receiving GEP testing among women with early-stage, HR+, HER2−, LN− breast cancer.

	Unadjusted	Adjusted
	OR (95% CI)	OR^a^ (95% CI)
Hospital location		
Urban	Ref	Ref
Large rural	**0.86 (0.74–0.99)**	1.05 (0.89–1.24)
Small rural	**0.45 (0.36–0.57)**	**0.55 (0.43–0.71)**
Age at diagnosis, years		
<50	Ref	Ref
50–59	0.98 (0.83–1.16)	1.04 (0.88–1.23)
60–69	**0.63 (0.54–0.74)**	**0.66 (0.56–0.78)**
≥70	**0.19 (0.16–0.22)**	**0.20 (0.17–0.24)**
Marital status		
Married/Living with partner	Ref	Ref
Other^b^/Unknown	**0.68 (0.62–0.76)**	**0.87 (0.77–0.97)**
Insurance status		
Insured^c^	Ref	Ref
Any Medicaid^d^	**1.27 (1.03–1.56)**	1.00 (0.80–1.26)
Uninsured/Unknown	0.91 (0.70–1.17)	0.78 (0.59–1.03)
Median household income		
<$50,000	Ref	Ref
$50,000-$59,999	**1.18 (1.03–1.34)**	1.15 (0.99–1.32)
$60,000-$69,999	**1.25 (1.08–1.44)**	**1.17 (1.01–1.37)**
≥$70,000	**1.32 (1.15–1.51)**	1.09 (0.94–1.32)
Year of diagnosis		
2010–2012	Ref	Ref
2013–2015	1.04 (0.92–1.17)	1.00 (0.87–1.14)
2016–2018	**1.43 (1.27–1.61)**	**1.47 (1.29–1.68)**
Histology		
Ductal	Ref	Ref
Lobular	1.05 (0.91–1.22)	1.15 (0.97–1.35)
Ductal + Lobular	**1.29 (1.01–1.64)**	1.29 (1.00–1.68)
Mucinous	**0.40 (0.29–0.54)**	**0.53 (0.38–0.74)**
NOS/Other	**0.54 (0.43–0.68)**	**0.61 (0.48–0.78)**
Tumor size, cm		
0.6–1.9	Ref	Ref
2.0–4.0	**1.34 (1.19–1.50)**	**1.29 (1.13–1.47)**
>4.0	**0.72 (0.54–0.97)**	**0.54 (0.39–0.75)**
Tumor grade		
Well differentiated	Ref	Ref
Moderately differentiated	**1.59 (1.43–1.77)**	**1.61 (1.43–1.81)**
Poorly differentiated/Undifferentiated	**1.49 (1.29–1.73)**	**1.32 (1.12–1.56)**
Unknown	0.84 (0.58–1.22)	0.79 (0.53–1.17)

Abbreviations: CI, confidence interval; CM, centimeter; GEP, gene expression profiling; HER2−, human epidermal growth factor receptor 2 negative; HR+, hormone receptor-positive; LN−, lymph node negative; NOS, not otherwise specified; OR, odds ratio; Ref, reference. Bold values indicate significance at *α* = 0.05. ^a^Adjusted for all other variables in the table. ^b^Divorced, separated, single, and widowed. ^c^ Private insurance and Medicare. ^d^ Indian Health Service. Bold values indicate significance at *α* = 0.05.

**Table 5:** Proportion with early-stage, HR+, HER2−, LN− female breast cancer receiving chemotherapy by ODX recurrence score.

	Hospital location					
	Urban		Large rural		Small rural	
	Total	Chemotherapy	Total	Chemotherapy	Total	Chemotherapy
	N	%	N	%	N	%
Test not done	2899	9.5%	483	7.5%	275	7.6%
Low risk	1441	1.9%	232	0.4%	68	1.5%
Intermediate risk	810	29.9%	101	24.8%	30	23.3%
High risk	194	83.5%	31	80.7%	7	84.7%

Abbreviations: HER2−, human epidermal growth factor receptor 2 negative; HR+, hormone receptor-positive; LN−, lymph node negative; ODX, Oncotype DX.

## Data Availability

The data that support the findings of this study are available from the ICR, but restrictions apply to the availability of these data, which were used under license for the current study, and so are not publicly available. Data are however available from the authors upon reasonable request and with permission of the ICR.
